# *PTEN* promoter methylation predicts 10-year prognosis in hormone receptor-positive early breast cancer patients who received adjuvant tamoxifen endocrine therapy

**DOI:** 10.1007/s10549-021-06463-6

**Published:** 2022-01-03

**Authors:** Yu Fan, Guiqin Xie, Zhu Wang, Yu Wang, Yanping Wang, Hong Zheng, Xiaorong Zhong

**Affiliations:** 1 Laboratory of Molecular Diagnosis of Cancer, West China Hospital, Sichuan University, Chengdu, 610041 People’s Republic of China; 2 Department of Head, Neck and Mammary Gland Oncology, Cancer Center, West China Hospital, Sichuan University, Chengdu, 610041 People’s Republic of China

**Keywords:** Breast cancer, PTEN, Tamoxifen, Promoter methylation, 10-year survival

## Abstract

**Purpose:**

There remain a lack of biomarkers for endocrine therapy resistance in patients with breast cancer (BC), which is proving to be a great challenge. In vitro experiments have shown that downregulation of PTEN expression leads to resistance to tamoxifen (TAM) in BC cells. We aimed to investigate the predictive role of tumor *PTEN* promoter methylation and PTEN expression in long-term survival after TAM adjuvant therapy in patients with early-stage BC.

**Methods:**

From 2001 to 2013, 105 patients with stage I–III BC who were treated with standardized adjuvant TAM for 5 years or until relapse in West China Hospital (WCH) were enrolled in this study. PTEN expression and DNA methylation of three specified sequences from the *PTEN* promoter in primary tumors were measured using immunohistochemistry and pyrosequencing. A cohort of 159 hormone receptor-positive patients receiving TAM treatment from The Cancer Genome Atlas (TCGA) database was used for verification.

**Results:**

Median follow-up time for the WCH cohort was 141.7 months. The low, moderate, and high PTEN expression groups had differing 10-year disease-free survival (DFS) (42.3%, 55%, 81%, respectively, *P* = 0.027) and overall survival (OS) rates (65%, 84.2%, 90.5%, respectively, *P* = 0.027). Higher methylation levels of the second sequence (− 819 to − 787 bp), rather than the first (− 1143 to − 1107 bp) or third sequence (− 663 to − 593 bp), independently increased the risk of disease recurrence (hazard ratio = 2.60) and death (hazard ratio = 3.79) in the WCH cohort, according to multivariate Cox regression analysis. Importantly, out of the five CpG islands located within this sequence, only high methylation of the − 796 CpG island predicted shorter DFS and OS. In TCGA validation cohort, there was also a trend of higher methylation of the − 796 CpG island correlating with shorter disease-free intervals, with borderline significance (*P* = 0.057).

**Conclusion:**

Low PTEN expression and high methylation of its promoter (sequence − 819 to − 787 bp) in tissue predict poor DFS and OS in hormone receptor-positive early BC patients who received adjuvant TAM.

**Supplementary Information:**

The online version contains supplementary material available at 10.1007/s10549-021-06463-6.

## Introduction

For patients with early-stage hormone receptor-positive (HR +) breast cancer (BC), 5 to 10 years of adjuvant hormone therapy can significantly slow down regional and remote recurrence and increase overall survival (OS) [1]. Tamoxifen (TAM) has been the mainstay of hormonal therapy, but resistance is common [2]. Researchers have made some achievements in unraveling the molecular biology related to endocrine disorders and resistance [3–5]. Estrogen receptor-alpha (ERα) loss by mutations, methylation, phosphorylation, truncated variants, and ER-associated transcription factors and co-activators are categorized as direct aberrations [6]. Although direct aberrations are a universal cause of TAM resistance, indirect causes such as alternative reproductive and survival stimuli also grant drug resistance to cancer cells.

PTEN is a major phosphatase that can halt the PI3K/AKT signaling pathway, one of the core cancer pathways [7]. Although PTEN can dephosphorylate lipids and proteins, it also has a separate role from that of a phosphatase under normal and pathological conditions. It also plays a role in genome stability and DNA repair [8]. It is subjected to translational positive and negative control, as well as posttranslationally through oxidation, phosphorylation, ubiquitination, and acetylation. Somatic mutations, deletion of gene loci, epigenetic silencing through promoter methylation, PTEN degradation, and post-translational alterations are speculated to be the main causes of PTEN inactivation [9]. DNA methylation is a biological process in which methyltransferases add methyl groups to DNA. This biological process only occurs in CpG dinucleotides [10]. Typically, the high incidence of hypermethylation of suppressor genes and hypomethylation of oncogenes makes methylation a promising biomarker and target for epigenetic therapy [11].

Although nearly half of patients with BC have reported loss of PTEN activity due to translational, genetic, or epigenetic changes, clinical evidence demonstrating the relationship between the prognosis of TAM and PTEN expression or its genetic/epigenetic regulation in patients with BC is limited and controversial [12]. A study indicated that decreased PTEN expression predicted relapse and poor prognosis in patients with BC treated with TAM [13]. Another scientific study showed that in patients with BC treated with TAM, there was a trend of increased risk for distant metastasis in patients with *PTEN* mutations compared with wild-type *PTEN* patients. However, this difference was not statistically significant (*P* = 0.19) [14].

Therefore, this study aimed to investigate whether *PTEN* promoter methylation level and PTEN expression in tumor tissue can predict long-term outcomes after TAM adjuvant therapy in patients with early breast cancer (EBC).

## Materials and methods

**Fig. 1:**
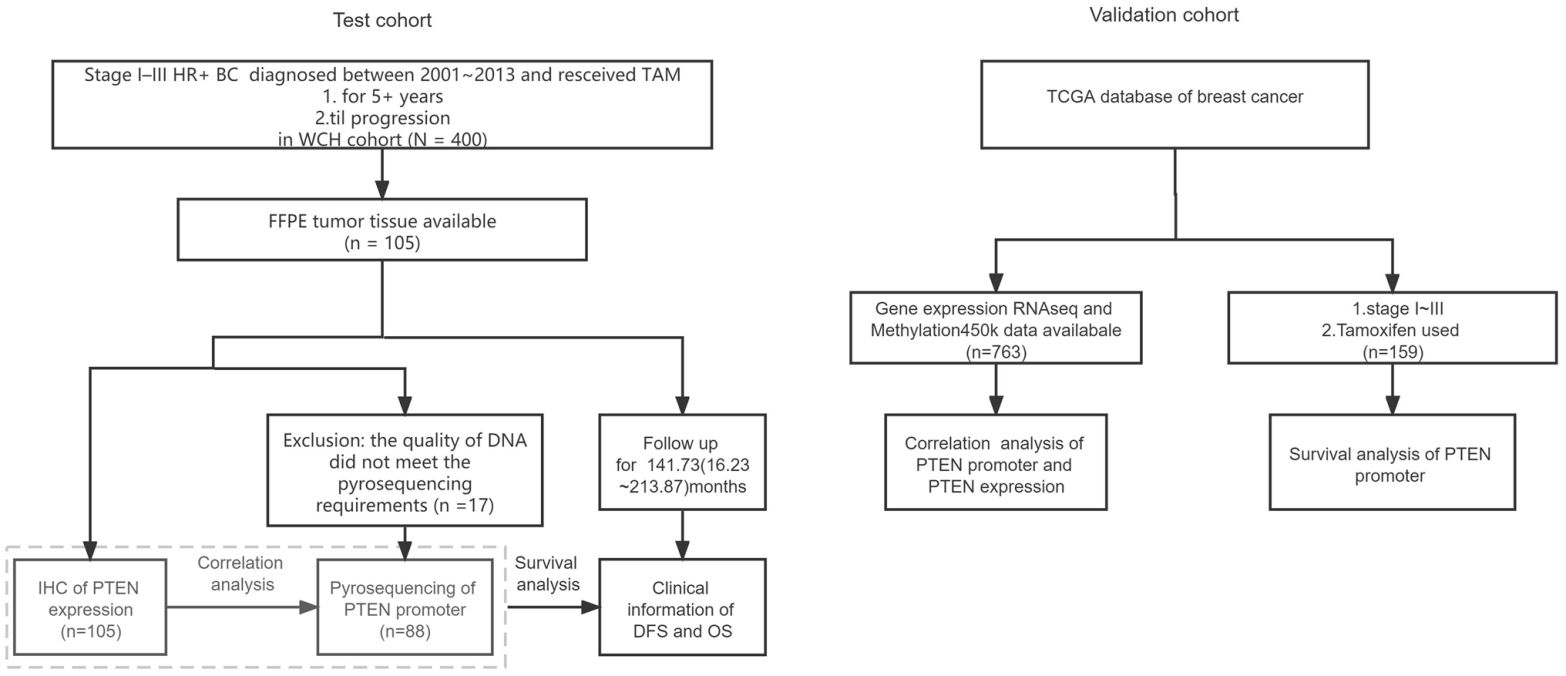
Flowchart of the study design and patient selection

A flowchart of the study design and patient selection is shown in Fig. 1.

### Patients

Patients with BC have been enrolled in the Breast Cancer Management Information System of the West China Hospital (WCH) of Sichuan University [15]. Professional physicians collected medical records, pathological diagnoses, and treatment information content. Every patient would have an outpatient or telephone follow-up every 3 to 4 months for 2 years, every 6 months for 3 to 5 years, and then every year after diagnosis. From March 2001 to September 2013, 400 women with HR + BC (stages I–III) accepted the standardized TAM treatment for 5 years or until recurrence. To prevent confounding effects, patients who received aromatase inhibitors (AI) or other endocrine therapies other than TAM were excluded. In addition, inclusion criteria included formalin-fixed paraffin-embedded tissue sections contained invasive cancer and the proportion of cancer cells was ≥ 80%. A total of 105 patients met this criterion and were included in the WCH cohort. Tumor samples from all 105 patients underwent immunohistochemistry (IHC), while those from 88 patients were available for pyrosequencing. The study was conducted under the Helsinki Declaration, and the West China Hospital Ethical Committee approved the study and granted exemption from the consent requirement. This study followed the reporting recommendations for tumor marker prognostic studies (REMARK) [16].

### Outcomes

Tumor recurrence, distant metastasis, and survival status were evaluated and confirmed by solid evidence of imaging or pathology. Disease-free survival (DFS) refers to the time from the beginning of TAM treatment to disease recurrence, death, or the latest follow-up. Overall survival (OS) refers to the time from the beginning of TAM treatment to death or the latest follow-up.

### Validation cohort of the Cancer Genome Atlas

The Cancer Genome Atlas (TCGA) database was downloaded as a verified set using the UCSC Xena browser (http://xena.ucsc.edu/public/), including information on *PTEN* methylation and RNA-seq, tumor stage, estrogen receptor (ER), progesterone receptor (PR), and human epithelial growth factor receptor 2 (HER2) status. DNA methylation profile in TCGA was measured using the Illumina Infinium HumanMethylation450 platform and was downloaded from “https://gdc-hub.s3.us-east-1.amazonaws.com/download/TCGA-BRCA.methylation450.tsv.gz.” DNA methylation levels, described as beta values, were collected for each array probe using the BeadStudio software. A total of 159 EBC patients treated with TAM alone or in combination were screened for analysis. Here, we used disease-specific survival (DSS) as the period from the date of initial diagnosis and treatment to the date of death or disease-free interval (DFI) as the period between a patient being declared disease free and first tumor development subsequent to this [17].

### IHC of PTEN

Based on the number of postoperative paraffin specimens, tissues were acquired from the pathology department of WCH. Three × 4 μm continuous formalin-fixed paraffin-embedded tumor slides were used for IHC and 1 × 4 μm slides were used for hematoxylin and eosin staining. The slides were deparaffinized and rehydrated in water. After blocking with 10% serum for 20 min at room temperature, the slides were incubated overnight at 4 °C with mouse anti-PTEN (dilution 1:150, DAKO) primary antibodies. Horseradish peroxidase-labeled secondary antibodies were added and incubated at room temperature for 30 min. The slides were then developed using the Dako REAL™ EnVision™ Detection System (DAKO Code K5007; Dako, Glostrup, Denmark). Negative and positive controls were given by replacing the primary antibody with PBS or mouse IgG2α. The immunostaining score (M-score) of PTEN, a semi-quantitative measure that weighs both positive cell proportion and staining intensity, was determined for each specimen by three pathologists [18]. Low M-score (< 16.67) was defined as low PTEN expression; moderate M-score (16.67 ≤ M-score < 33.33) was defined as moderate PTEN expression; and high M-score (≥ 33.33) was defined as high PTEN expression. Supplementary Fig. 1 shows the immunohistochemical results of low, moderate, and high PTEN expression and negative controls.

### Pyrosequencing of the *PTEN* promoter

Serial Sects. 120 μm thick were used for pyrophosphate sequencing. Paraffin-embedded tissue Sects. (10-μm-thick slices) were obtained from 88 patients who passed quality control. Genomic DNA was converted to bisulfite. Three target sequences, seq1 (− 1143 to − 1107 bp), seq2 (− 819 to − 787 bp), and seq3 (− 663 to − 593 bp) upstream from the *PTEN* transcription start site (TSS) were amplified by PCR. Next, a pyrosequencing detector (PyroMark Q96 ID, QIAGEN) and Pyro Q-CpG software were used to analyze the methylation status of each CpG site. *PTEN* DNA promoter seq1, seq2, and seq3 contained four, five, and eight CpG sites, respectively (Fig. 3a). The different primers used for the three sequences are listed in Supplementary Table 1.

### Statistical analysis

Survival analysis was performed using the Kaplan–Meier method. Spearman’s rho was used for the correlation of PTEN expression and promoter methylation analysis. Cox’s proportional hazards model was used to analyze survival (time to event) outcomes on one or more predictors. The Fisher’s exact test and Mann–Whitney U test were used for group comparison analysis. The Survminer R package (https://github.com/kassambara/survminer) was employed to identify the optimal cut-off point for high risk and low-risk groups of PTEN expression and methylation levels of CpG islands. Statistical significance was set at *P* < 0.05.

## Results

### Patient characteristics

The detailed clinical pathological characteristics of the WCH and TCGA cohorts are summarized in Table 1. The mean ages were 44.8 ± 8.69 years and 52.64 ± 13.69 years in WCH and TCGA cohorts, respectively. Menopause rates were 15.23% and 45.28%, respectively. In the WCH group, 94.29% of patients received chemotherapy, whereas 68.55% of patients in the TCGA cohort received chemotherapy. During the median follow-up time of 141.7 (16.2–213.9) months in WCH, 45 (42.8%) patients had a recurrence after surgery and 28 (26.7%) patients died. The follow-up time in the TCGA cohort was 36.7 (5.3–163.1) months.

**Table 1:** Clinical and pathological features of 105 tamoxifen-treated invasive breast cancer patients of WCH and 159 tamoxifen-treated patients from TCGA

Features	WHC cohort (*n* = 105)	TCGA cohort (*n*= 159)	*P*
	Cases (percentage)	Cases (percentage)	
Age at diagnosis(years) mean ± SD	44.8 ± 8.69	52.64 ± 13.69	0.00
Menopause			0.00*
Yes	16(15.23%)	72(45.28%)	
No	89(84.76%)	80(50.32%)	
N/A	0	7(4.40%)	
Clinic stage			0.10
I	20(19.04)	19(11.95%)	
II	51(48.57)	97(61.01%)	
III	31(29.52)	42(26.42%)	
N/A(no metastatic)	3(2.85)	1(0.63%)	
HR			0.001*
ER (+)/PR (+)	83(79.05%)	136(85.53%)	
ER (+)/PR (−)	10 (9.52%)	21(13.21%)	
ER (−)/PR (+)	12 (11.43%)	2(1.26%)	
Her2			0.06
Positive	7(6.67%)	25(15.72%)	
Negative	92(87.6%)	129(81.13%)	
Uncertain	6(5.71%)	5(3.14%)	
Chemotherapy			0.00*
Yes	99(94.29%)	109(68.55%)	
No	6(5.71%)	50(31.45%)	
Radiotherapy			0.12
Yes	53(50.48%)	95(59.75%)	
No	52(49.52%)	62(38.99%)	
Unknown	0	2(1.26%)	
Median follow-up, range (months)	141.73(16.23–213.87)	36.7(5.33–219.77)	0.00*

* P < 0.05

### PTEN expression and prognosis

**Fig. 2:**
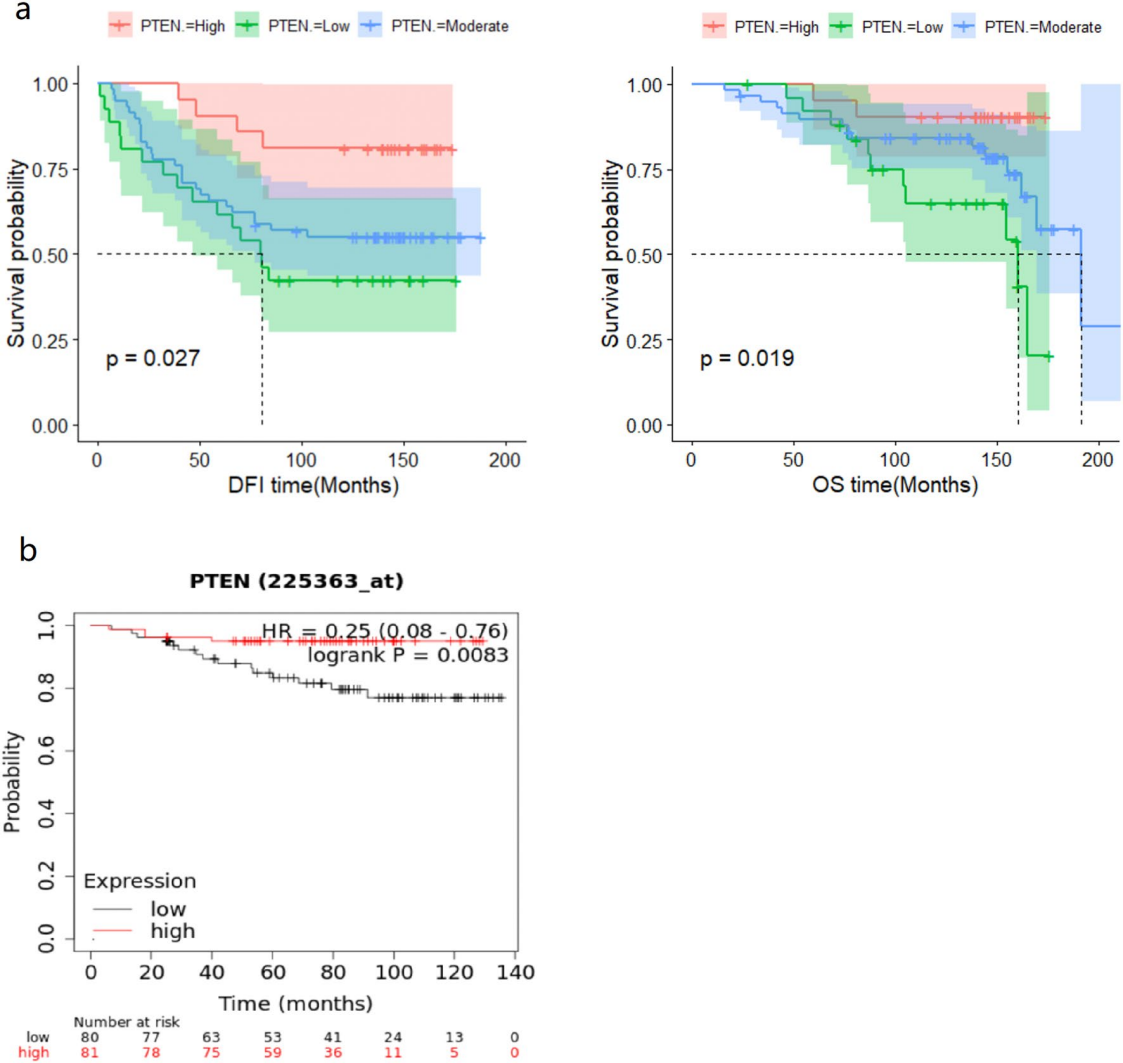
Outcome of tamoxifen-treated patients with different PTEN expressions. **a** Kaplan–Meier curve of disease-free survival (left) and overall survival (right) for PTEN low, moderate, and high expression patients who received adjuvant tamoxifen treatment. **b** Kaplan–Meier curve of RFS of PTEN low and high expression patients, using an online method that combined public expression databases. Inclusion criteria were tamoxifen-treated only, non-metastatic, and estrogen receptor-positive breast cancer patients (http://kmplot.com)

In the WCH cohort, 26, 58, and 21 patients were classified as low, moderate, and high PTEN expression groups, respectively, according to the M-score. Ten-year DFS rates for the low, moderate, and high PTEN expression groups were 42.3%, 55%, and 81%, respectively (log rank *P* = 0.027; Fig. 2a). Univariate Cox regression analysis showed that PTEN expression was a protective factor against relapse after TAM treatment (hazard ratio [HR] 0.55, 95% CI 0.36–0.86, *P* = 0.009). After adjusting for stage, menopausal status, radiotherapy, and HER2 status, PTEN expression was still a protective factor against relapse (adjusted HR 0.51, 95% CI 0.32–0.8, *P* = 0.004).

More importantly, 10-year OS rates for the low, moderate, and high expression groups were 65%, 84.2%, and 90.5%, respectively (log rank *P* = 0.027; Fig. 2a). Univariate Cox regression analysis showed that PTEN expression was also a protective factor against death after TAM treatment (HR 0.44, 95% CI 0.24–0.8, *P* = 0.007) (Table 2). After adjusting for stage, menopause, and radiotherapy, PTEN expression was still a protective factor from death (HR 0.49, 95% CI 0.27–0.88, *P* = 0.02) (Table 2).

**Table 2:** Univariate and multivariate cox regressions of PTEN expression for DFS and OS

	DFS HR 95%CI		DFS HR 95%CI		OS HR 95%CI		OS HR 95%CI	
	Univariate Cox Regression	*P*	Multivariate Cox Regression	*P*	Univariate Cox Regression	*P*	Multivariate Cox Regression	*P*
Age	0.99 (0.95–1)	0.53			0.98 (0.93–1)	0.34		
Stage	2.6 (1.6–4.2)**	0.000	2.31 (1.434–4.01)**	0.003	2.7 (1.4–5)*	0.0021	2.16 (1.13–4.15)*	0.02
ki67	1.2 (0.59–2.3)	0.68			0.9 (0.37–2.2)	0.81		
HER2	2.9 (1.1–7.4)*	0.03	4.25 (1.55–11.62)**	0.005	2.6 (0.77–8.7)	0.13		
Chemo	7.5e+07 (0-Inf)	1			7.4e+07 (0-Inf)	1		
Radio	2.4 (1.3–4.5)**	0.005	1.47 (0.68–3.17)	0.323	3.2 (1.4–7.6)*	0.008	1.74 (0.71–4.33)	0.23
Menopause	2.2 (1.1–4.4)*	0.021	1.86 (0.9–3.85)	0.095	2.8 (1.1–6.7)*	0.024	2.3 (0.93–5.69)	0.07
PTEN(M-score)	0.55(0.36–0.86)*	0.009	0.51 (0.32–0.8)*	0.004	0.44 (0.24–0.8)*	0.007	0.49 (0.27–0.88)*	0.02

For multivariate cox regression, stage, menopausal status, radiotherapy, and HER2 status were included.*P < 0.05, **P < 0.01

The relationship between PTEN expression and survival was confirmed using an online method (http://kmplot.com). A PostgreSQL server, which integrates gene expression and clinical data from Genome Expression Omnibus, European Genome-phenome Archive, and TCGA simultaneously, handles the database [19]. A total of 161 non-metastatic, HR+ patient treated with TAM only were extracted. The hazard ratio of recurrence-free survival (RFS) was 0.25 (log rank *P* = 0.008) for patients with high PTEN expression (Fig. 2b).

### *PTEN* promoter methylation and prognosis

**Fig. 3:**
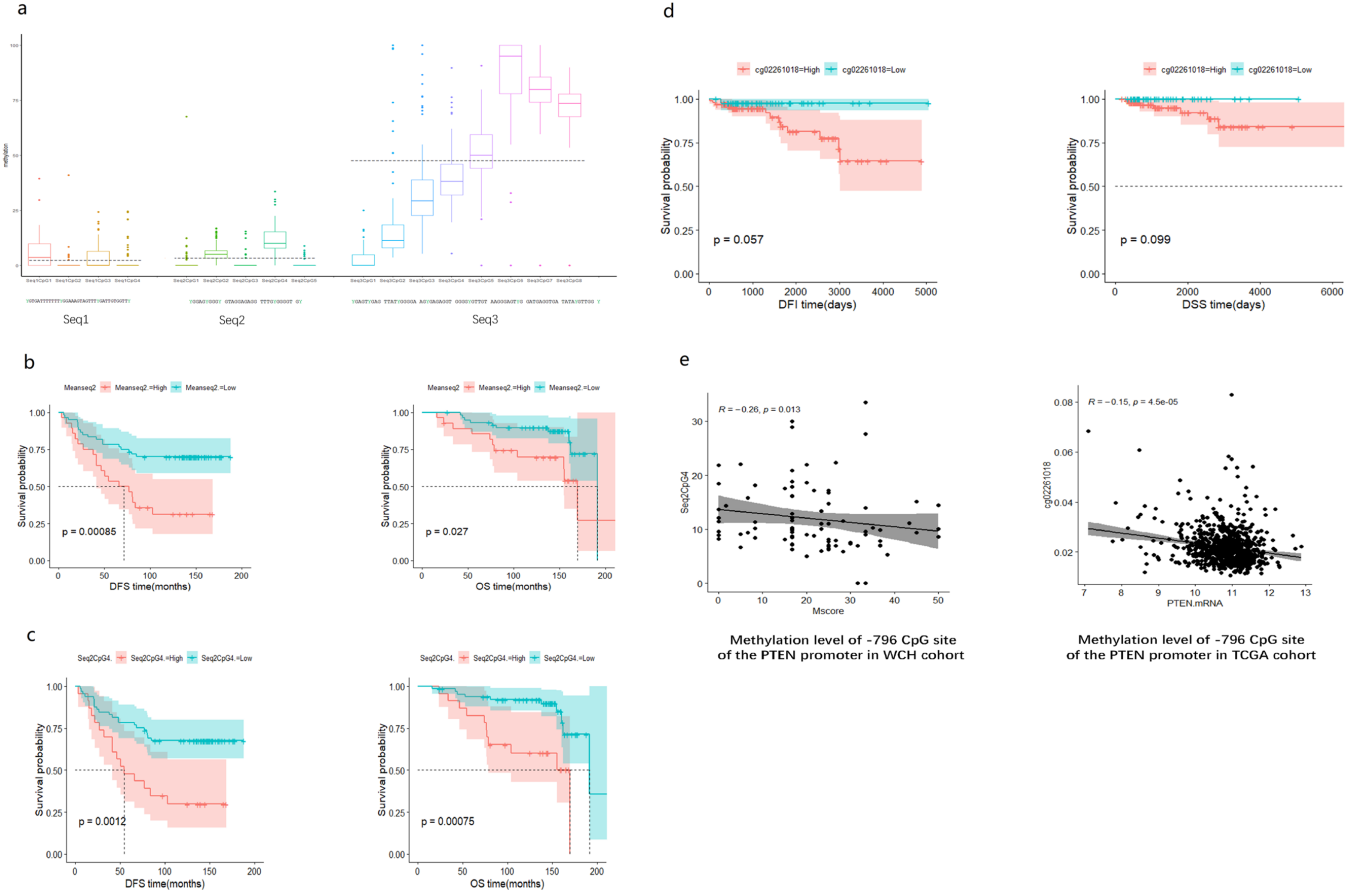
The impact of PTEN promoter methylation on outcome of TAM-treated EBC patients and PTEN expression. **a** Boxplot of methylation data of 17 CpG sites in 3 sequences from − 1143 to − 593 bp on the *PTEN* promoter in 88 samples using pyrosequencing. Dotted lines demonstrate the average methylation level of seq1 (− 1143 to − 1107 bp), seq2 (− 819 to − 787 bp), and seq3 (− 663 to − 593 bp). The lower panel shows the sequences of these 3 regions. **b** The Kaplan–Meier curve of disease-free survival (left) and overall survival (right) of patients with higher and lower methylation levels of the − 819 to − 787 bp sequence in the WCH cohort. **c** The Kaplan–Meier curve of disease-free survival (left) and overall survival (right) of patients with higher and lower methylation levels of the − 796 CpG site in the WCH cohort. **d** The Kaplan–Meier curve of disease-free interval (left) and overall survival (right) of patients with higher and lower methylation levels of the − 796 CpG site in TCGA cohort. **e** The correlation of the methylation level of the − 796 CpG site and the PTEN expression in WCH (left) and TCGA (right) cohorts. In the WCH cohort, M-score represents PTEN protein expression. In TCGA cohort, transformed RSEM normalized count represents *PTEN* mRNA level

Using pyrosequencing, we successfully obtained the methylation data of 17 CpG sites in the three sequences defined in Sect. 2.5 in 88 samples (Fig. 3a). The average methylation levels of seq1, seq2, and seq3 were 3.04 (range: 0–15.06), 4.03 (range: 0–19.59), and 47.7 (range: 2.14–88.16), respectively, as demonstrated in Fig. 3a. We found that the mean methylation level of seq2 was associated with the outcome of TAM-treated patients (Table 3, Fig. 3b), rather than seq1 or seq3 (Supplementary Fig. 2). The Survminer R package was used to determine the optimal cut-off point for each variable. When we used ≥ 4.82 as the cut-off for mean seq2 methylation level, the median DFS for high and low methylation groups was 66.1 months vs not reached (NR) (*P* = 0.0009) and OS was 169 vs. 191 months (*P* = 0.027). The high-methylated seq2 group had a higher hazard ratio for relapse (HR 2.9, 95% CI 1.5–5.5, *P* = 0.001) and death (HR 2.6, 95% CI 1.1–6, *P* = 0.030). After adjusting for stage, radiotherapy, and HER2 expression, methylation of seq2 was still a risk factor for relapse (HR 2.6,95% CI 1.27–5.33, *P* = 0.009) and death (HR 3.79, 95% CI 1.41–10.26, *P* = 0.008).

**Table 3:** Univariate and multivariate cox regressions of mean methylation level of the 3 sequences for DFS and OS

Mean methylation	DFS HR 95%CI		DFS HR 95%CI		OS HR 95%CI		OS HR 95%CI	
Level	Univariate Cox Regression	*P*	Multivariate Cox Regression	*P*	Univariate Cox Regression	*P*	Multivariate Cox Regression	*P*
Seq1	1.6 (0.76–3.3)	0.22			2 (0.77–5.3)	0.15		
Seq2	2.9 (1.5–5.5)**	0.001	2.60 (1.27–5.33)**	0.009	2.6 (1.1–6)*	0.033	3.79(1.41–10.26)**	0.008
Seq3	0.54 (0.26–1.1)	0.093			0.38 (0.14–1.1)	0.07		

For multivariate cox regression, stage and HER2 status and radiotherapy were included. *P < 0.05, **P < 0.01

Next, we performed a survival analysis of single CpG island methylation within seq2. Using 15.16 as the cut-off point, patients with a higher methylated − 796 CpG island showed shorter DFS (54.8 months vs NR) (*P* = 0.001) as well as shorter OS (160 months vs. 191 months) (*P* = 0.001) than those with lower methylation. Higher methylation of − 796 CpG island predicted a higher risk of relapse (HR 2.8, 95% CI 1.5–5.4, *P* = 0.002) and death (HR 4.1, 95% CI 1.7–9.9, *P* = 0.002). Multiple Cox regression analysis demonstrated that the -796 site methylation level was an independent predictor of DFS (HR 5.99, 95% CI 2.22–16.16, *P* = 0.0004) after adjusting for stage, HER2, and radiotherapy and OS (HR 6.2, 95% CI 2.32–16.33, *P* = 0.0003) after adjusting for stage or HER2 status (Table 4). However, the methylation levels of the other four CpG islands were not significant. Spearman’s rho test showed that the methylation level of the − 796 CpG island was correlated with the M-score of PTEN expression (correlation coefficient = − 0.26, *P* = 0.013) (Fig. 3e).

**Table 4:** Univariate and multivariate cox regressions of 5 singe CpG methylation level of the second sequence for DFS and OS

Single CpG methylation of Seq2	DFS HR 95%CI		DFS HR 95%CI		OS HR 95%CI		OS HR 95%CI	
	Univariate Cox Regression	*P*	Multivariate Cox Regression	*P*	Univariate Cox Regression	*P*	Multivariate Cox Regression	*P*
CpG1	1.8 (0.92–3.7)	0.082			2.4 (0.94–6.1)	0.068		
CpG2	1.7 (0.86–3.3)	0.13			1.9 (0.82–4.6)	0.13		
CpG3	2 (0.77–5.1)	0.16			1.6 (0.37–7.2)	0.51		
CpG4	2.8 (1.5–5.4)**	0.002	5.99 (2.22–16.16)**	0.0004	4.1 (1.7–9.9)**	0.002	6.20 (2.32–16.63)**	0.0003
CpG5	2.2 (0.97–5)	0.061			1.2 (0.33–4.1)	0.82		

For multivariate cox regression, stage and HER2 status and radiotherapy were included. *P < 0.05, **P < 0.01

### TCGA validation of -796 CpG island and prognosis

To verify the prognostic role of *PTEN* promoter methylation, 159 HR + EBC patients treated with either TAM alone or in combination were enrolled for analysis. The corresponding site of -796 on the Illumina Infinium HumanMethylation450 platform was labeled as cg02261018. We found that the higher the beta value of cg02261018, the shorter the DFI time with borderline significance (*P* = 0.057). DSS time was not significantly different in this cohort (*P* = 0.099) (Fig. 3d).

In the TCGA cohort, which contained 763 patients with the beta value of the cg02261018 CpG island available, an association between *PTEN* mRNA expression and cg02261018 methylation was also detected (correlation coefficient = − 0.15, *P* = 0.00005) (Fig. 3e).

## Discussion

In the test cohort of WCH HR+EBC adjuvant TAM-treated patients, we found that the higher methylation level of the − 819 to − 787 sequence of the *PTEN* promoter was an independent predictor of outcome, including DFS and OS. More importantly, our results indicate that the methylation level of a specified CpG island (− 796) within this sequence was negatively correlated with the prognosis of TAM-treated EBC patients and PTEN expression. These results were verified using a public database.

Much uncertainty still exists regarding the relationship between *PTEN* methylation and TAM resistance. Our study provides important clinical evidence for long-term follow-up. It was previously shown that the proportion of *PTEN* methylation was significantly higher in serum than in normal tissues and it is closely correlated with tumor tissues [20]. Nevertheless, tumor suppressor genes, including *PTEN*, were characterized by a low (< 1%) average methylation level and a low mean epimutation rate (< 0.0001% to 0.1%) [21]. According to Phuong et al., aberrant methylation of the *PTEN* promoter was caused by SAM increase with DNMT1 overexpression in a TAM-resistant cell line and proposed its therapeutic target potential. They also used methylation-specific PCR to show that two sites within the *PTEN* promoter were methylated in this TAM-resistant cell line. This caused lower expression of PTEN and upregulation of Akt phosphorylation in vitro and in an animal study [22]. We evaluated the clinical prognostic role of PTEN and methylation of 17 single sites in patients registered in the Breast Cancer Information Management System at the WCH. In our study, single CpG hypermethylation was significantly more frequent in position − 663 to − 593 bp of *PTEN* in breast cancer tissue, but the methylation level did not correlate with the outcomes of patients. Conversely, the hypomethylated sequence − 819 to − 787 showed an association with long-term DFS and OS. However, the CpG sites located on this sequence were all hypomethylated. The quantitative method for methylation determination in TCGA is the Illumina HumanMethylation450 BeadChip, whereas we used pyrosequencing, which is considered the current ‘gold standard’ for DNA methylation analysis at single-nucleotide resolution [23]. Our study suggests that this − 796 locus methylation may be a potential marker of endocrine therapy resistance.

The mechanisms underlying the regulation of prognosis by *PTEN* methylation may be its direct influence on PTEN expression. Our results suggest a connection between − 796 CpG island methylation and PTEN expression, based on IHC, and this connection was verified using the TCGA database. In animal models of HR+BC, periodic fasting or a fasting-mimicking diet intensified the curative effects of TAM by increasing PTEN expression [24]. A retrospective study comprising 49 ER- and/or PR-positive patients with primary BC reported that reduced PTEN expression led to shorter relapse-free survival of TAM-treated patients, and loss of heterozygosity (LOH) of PTEN was significantly associated with shorter disease-free survival, cancer-specific survival, and OS of TAM-treated patients with BC [25]. Another retrospective study analyzed 78 postmenopausal stage I/II patients with BC treated with adjuvant TAM and found that patients with PTEN-negative BC had significantly shorter DFI and OS compared to PTEN-positive patients with BC [26].

Our results were consistent with those of these studies. In our study, the recurrence and death risks were reduced by 49% and 51% in the high PTEN expression group, respectively. This suggests that PTEN expression may be a protective factor against relapse after TAM treatment. A total of 161 non-metastatic, HR+ patients treated with TAM only were extracted from databases of publicly released studies. Consistantly, the risk of relapse was decreased by 75% in patients with high PTEN expression, compared to those with low expression.

There were some limitations to our study. First, all subjects were from a single center. Second, pyrosequencing only covered the partial promoter region of *PTEN* and there were some additional CpG islands that need to be explored.

In conclusion, low PTEN expression and high methylation of its promoter (sequence − 819 to − 787 bp) in tumor tissue predict poor DFS and OS in HR+EBC patients who received adjuvant TAM endocrine therapy. In particular, the methylation status of the − 796 site has the potential to predict the prognosis of endocrine therapy.

## Supplementary Information

Below is the link to the electronic supplementary material.

Supplementary file1 (PDF 391 kb)

## Data Availability

The data that support the findings of this study are available from the corresponding author X.R Zhong upon reasonable request.
