# Supplementary material for: PTEN promoter methylation predicts 10-year prognosis in hormone receptor-positive early breast cancer patients who received adjuvant tamoxifen endocrine therapy
Source: Breast Cancer Res Treat. 2022 Jan 3;192(1):33–42. doi: 10.1007/s10549-021-06463-6 (PMC8841328; doi:10.1007/s10549-021-06463-6)
Supplement: Supplementary file 1 — Supplementary file1 (PDF 391 kb) [file 10549_2021_6463_MOESM1_ESM.pdf]

Fig. 1

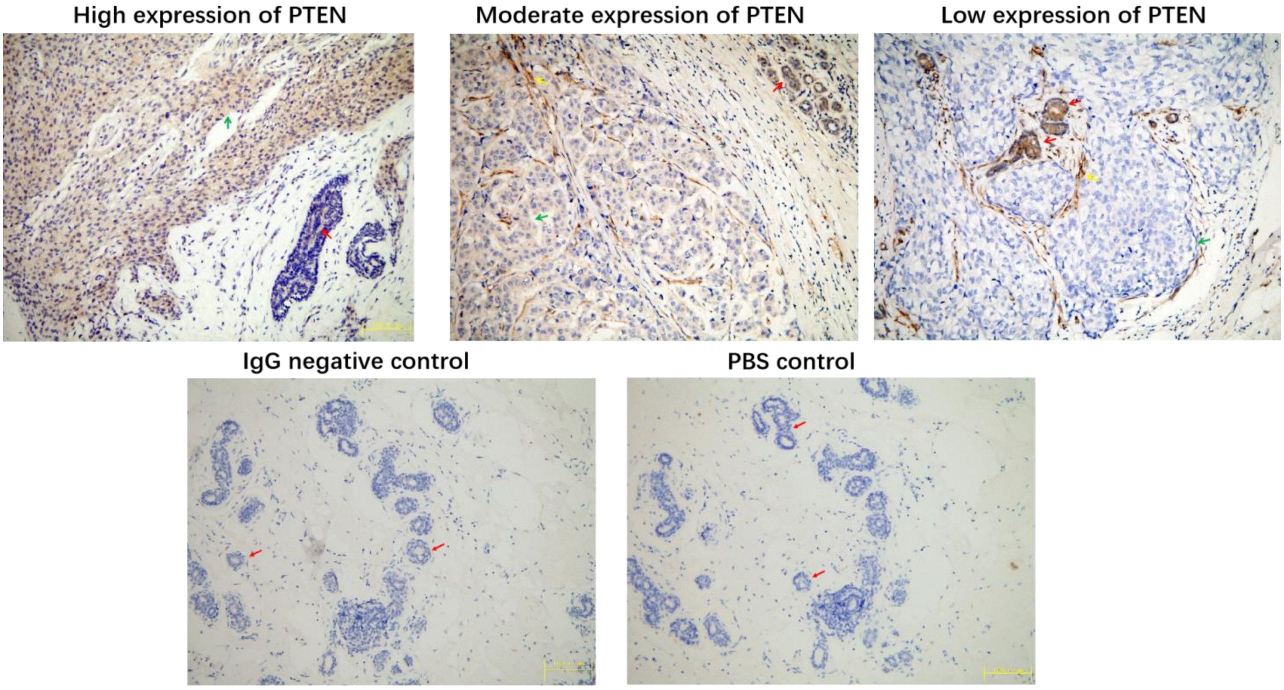

Supplementary Fig. 1

The immunohistochemical results showed low, moderate, and high expression of PTEN and negative IgG and PBS controls. The red arrows indicate normal ducts (or ducts with mild breast hyperplasia). The yellow arrows indicate staining of endothelial or nerve cells, and the green arrows indicate staining of neoplastic cells, infiltrating ductal carcinoma or ductal carcinoma *in situ*. The surrounding normal epithelium serves as an internal control (red arrows).

Fig. 2

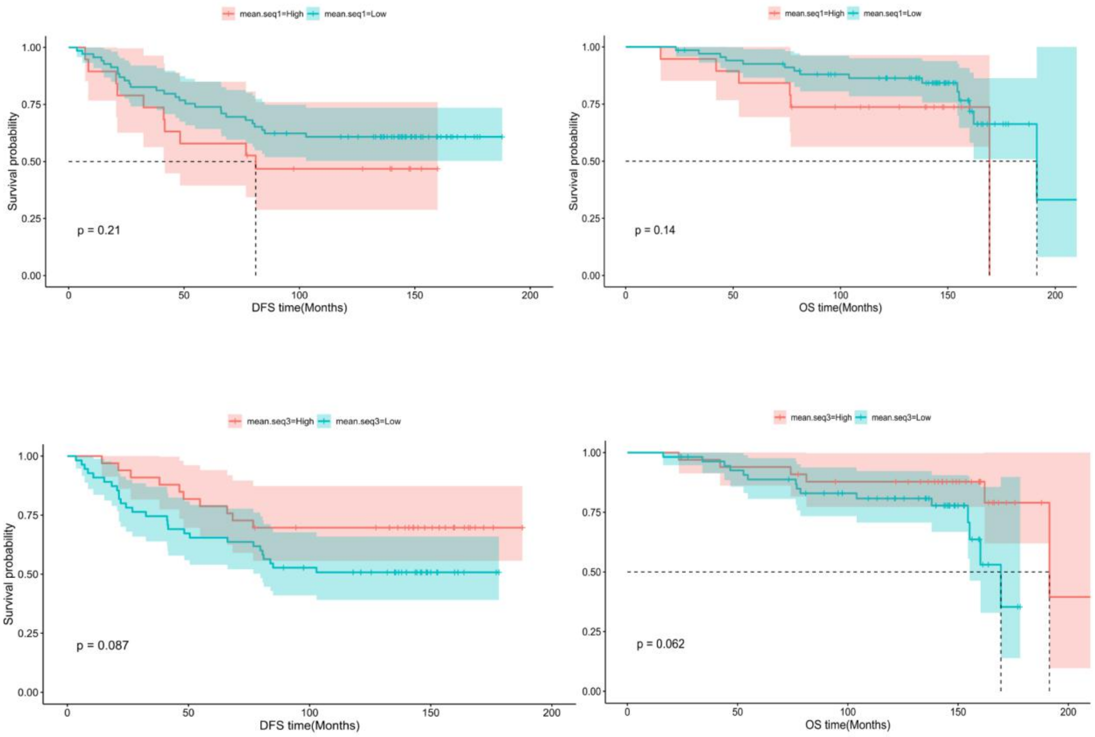

Fig. 2 The Kaplan–Meier curve of DFS time (left) and OS time(right) for patients with higher and lower mean methylation level of -1143 to -1107 sequence(upper) and -819 to -787 sequence(lower) in WCH cohort.

Table 1 Primers for PCR amplification and sequencing

Seq name	location	Primer	sequence
Seq1	-1143 to -1107	Forward primer	5'- GGGAGTGGGAATTTGGAAAGTTT -3'
		Reverse primer	5'- Biotin-AACTCCATTCTCAAAAACCACCTAACC -3'
		Sequencing primer	5'- GGAAAGTTTTTTAATTAGGGATAT-3'
Seq2	-819 to -787	Forward primer	5'- TTGGAGTTAGAGGGGAAAGATGT-3'
		Reverse primer	5'- Biotin- TTCAAAAACCCAAAAAACACCTATCTA-3'
		Sequencing primer	5'- GTTAGATTTTTTTGGGGGTAT -3'
Seq3	-663 to -593	Forward primer	5'- GGTGTTTTTTGGGTTTTTGAAAT-3'
		Reverse primer	5'-Biotin-TTCCCCCAAATCTATATCCTCATAATATCA -3'
		Sequencing primer	5'- GAGAGTTTTTATTTTTAGGGTAA -3'
